# Evaluation of the impacts of a district-level mental health care plan on contact coverage, detection and individual outcomes in rural Uganda: a mixed methods approach

**DOI:** 10.1186/s13033-019-0319-2

**Published:** 2019-09-30

**Authors:** J. E. M. Nakku, S. D. Rathod, E. C. Garman, J. Ssebunnya, S. Kangere, M. De Silva, V. Patel, C. Lund, F. N. Kigozi

**Affiliations:** 1 Makerere University College of Health Sciences/Butabika National Referral and Teaching Mental Hospital, Kampala, Uganda; 2 Department of Population Health, London School of Hygiene & Tropical Medicine, London, UK; 3 Alan J Flisher Centre for Public Mental Health, Department of Psychiatry and Mental Health, University of Cape Town, Cape Town, South Africa; 4 Makerere University/Butabika National Referral and Teaching Mental Hospital, Kampala, Uganda; 5 The Wellcome Trust, London, UK; 6 Department of Global Health and Social Medicine, Harvard Medical School, Boston, MA USA; 7 Department of Global Health and Population, Harvard TH Chan School of Public Health, Boston, MA USA; 8 Centre for Global Mental Health, Health Services and Population Research Department, Institute of Psychiatry, Psychology and Neurosciences, King’s College London, London, UK

## Abstract

**Background:**

The burden of mental disorders in low- and middle-income countries is large. Yet there is a major treatment gap for these disorders which can be reduced by integrating the care of mental disorders in primary care.

**Aim:**

We aimed to evaluate the impact of a district mental health care plan (MHCP) on contact coverage for and detection of mental disorders, as well as impact on mental health symptom severity and individual functioning in rural Uganda.

**Results:**

For adults who attended primary care facilities, there was an immediate positive effect of the MHCP on clinical detection at 3 months although this was not sustained at 12 months. Those who were treated in primary care experienced significant reductions in symptom severity and functional impairment over 12 months. There was negligible change in population-level contact coverage for depression and alcohol use disorder.

**Conclusion:**

The study found that it is possible to integrate mental health care into primary care in rural Uganda. Treatment by trained primary care workers improves clinical and functioning outcomes for depression, psychosis and epilepsy. Challenges remain in accessing the men for care, sustaining the improvement in detection over time, and creating demand for services among those with presumed need.

## Introduction

There is increasing recognition of the growing prevalence and impact of mental illness in low and middle income countries (LMICs). Current estimates suggest that worldwide, mental illness accounts for 32.4% of years lived with disability (YLDs), and 13% of disability-adjusted life years (DALYs) [1]. Of the DALYs contributed by mental, neurological and substance use disorders, depression alone contributes 40.5% and is projected to be the second leading cause of burden of disease globally by 2020 [2]. The prevalence of mental disorders in Uganda is believed to be high. Ovuga et al. [3] found the prevalence of depression in two rural districts in the north and eastern parts of Uganda to be 17.4%. Data regarding the treatment gap in Uganda is lacking but it is estimated to be as large as 85% [4].

Primary care plays a pivotal role in treating mental health conditions. Many patients with symptoms of mental distress are primarily and often exclusively seen by general practitioners in primary health care settings [5]. Hence, the World Health Organization (WHO) recommends the integration of care of mental health conditions into primary care by shifting care from specialist (i.e. psychiatrists, psychiatric nurses, psychologists) to generalist providers. The integration of mental health into primary health care enhances access, promotes respect of human rights, is affordable and cost effective and generates good health outcomes [6].

Emerging evidence shows the effectiveness and cost-effectiveness of treatment provision by generalist providers (e.g. nurse, general practitioner, community health worker) for mental, neurological and substance use disorders such as depression, alcohol use disorder, psychosis and epilepsy [7]. Previous studies have shown that complex interventions that incorporated clinician education, an enhanced role of the nurse (nurse case management), and a greater degree of integration between primary and secondary care were effective in improving patient outcomes [8]. This happens through increased contact with, as well as detection and treatment by trained primary care providers. However, data are lacking about the feasibility and impact of integrating mental health treatments into primary care at a system level in LMICs. The aim of this study is to report the impact of implementing a novel district mental healthcare plan on contact coverage, case detection, and individual level clinical and functioning outcomes in a rural African setting: the Kamuli district in Uganda.

## Methods

### Setting

The PRogram for Improving Mental health carE (PRIME) research consortium aimed to implement and evaluate mental health care plans for adults in five LMICs districts including Kamuli District, Uganda [9]. The others countries are India, South Africa, Nepal and Ethiopia. Details of the demographic characteristics of Kamuli have been previously published in the district’s situation analysis [10].

Kamuli district is a small rural district in eastern Uganda covering 1557 km^2^ of the 241,000 km^2^ of the entire country. The district has a population of 490,000 which is predominantly (97%) rural with inhabitants typically occupied as subsistence farmers, fishermen and small traders. It has a population density of 321.6/km^2^ which makes it one of the most densely populated districts in the country. The population is largely poor with a literacy rate of 55%, which is lower than the national average estimated at 73.8% in 2015. Birth rate in Kamuli district is 6.4 children per woman of child bearing age which is higher than the national average of 5.2 [11].

Kamuli is geographically divided into 2 counties, each with at least 4 sub counties. The health system arrangement mirrors this geographical division. At each county there is a level four public health center (HCIV) which operates like a mini hospital with a theatre, a maternity unit and admission beds. A HCIV facility is a 30–40 bed unit that serves 100,000 population and is staffed with a doctor, nurses, nursing assistants and other support staff such as records personnel and security officers. At every sub county there is a public health center level 3 (HCIII) which is primarily an outpatient primary care unit with only maternity beds serving a population of about 20,000 people [12]. It is managed by a clinical officer (physician assistant) and midwives. A level 2 health centre (HCII) is a small dispensary serving at least 5000 people that is managed by a general nurse who handles common patient ailments such as fevers, cough, influenza and wounds. The district is also served by 2 general hospitals serving up to 500,000 population. The general hospitals serve as the referral centers for the lower health facilities. The district is served by a regional referral hospital in the neighboring district of Jinja, located 62 km away from Kamuli town. The regional referral hospital has 30 psychiatric beds and is staffed with specialist doctors such as physicians, surgeons, gynecologists and psychiatric clinical officers to take care of patients needing specialized care.

### Implementation

In partnership with the Uganda ministry of Health and informed by WHO’s mental health gap action program (mhGAP) implementation guidelines, the PRIME-Uganda team developed, implemented and evaluated a mental healthcare plan (MHCP) for Kamuli District.

The MHCP was developed using a theory of change framework [13, 14] with involvement of a number of stakeholders, including health workers, health managers, political leaders, service users and their carers, as well as lay opinion leaders in the district. The MHCP comprised 5 packages of care namely: (i) awareness raising and knowledge enhancement, (ii) detection, (iii) treatment, (iv) recovery and (v) program management. Each package of care comprised several components that informed the activities under each package. These are summarized in Table 1. Implementation of the MHCP happened at three levels of the district health care system, namely the district, health facility and community levels. The Kamuli MHCP and its evaluation methods have been previously described [15, 16]. The implementation and evaluation timelines are summarised in Additional file 1: Figure S1.

**Table 1:** Components of the mental health care plan in Kamuli District, Uganda

Package area	Awareness and knowledge enhancement	Detection	Treatment	Recovery	Program management
Level					
Health organisation	Engagement Advocacy Mental health literacy				Drug supply chain management HMIS Human resource support, motivation and supervision Capacity-building Routine monitoring and evaluation
Primary Health care facility	Standardized In-Service training	Screening and assessment	Psychotropic medication Basic psychosocial support		
Community	Community sensitisation/anti-stigma/mobilisation Training of Village Health Team workers	Community detection		Outreach and adherence support Community Based Rehabilitation (CBR)	

### Study designs

The PRIME evaluation methods were developed to assess the effect of the MHCP on the following three sequential stages to ultimately reduce the treatment gap: (1) whether adults with probable depression or Alcohol Use Disorder (AUD) in the population make contact with a primary care provider, (2) whether adults presenting with probable depression or AUD who make contact with a primary care provider are detected, and (3) whether adults who initiate treatment for depression, psychosis or epilepsy in primary care clinics achieve reduction in their symptom severity and functional impairment. In an area where mental health care was absent prior to the implementation of the MHCP, changes in these outcomes are interpreted as evidence of the MHCP’s effect. The aims, design, and measures of the three studies have been previously described in detail [16–19], and are summarized below.

#### Aim1: contact coverage for depression and AUD

The community study aimed to estimate the change in population-level contact coverage. Contact coverage was defined as the proportion of adults who have probable depression and probable AUD who contacted a primary care provider. Two population-based cross-sectional surveys with independent samples were conducted. The baseline community study round (n = 1290) was conducted from May to June 2013, 3 months before implementation of the MHCP and the follow up (n = 3481) from August 2016 to March 2017, at least 36 months after the start of implementation. Using random selection at each stage, villages were selected in Kamuli District, then households within villages, then one available adult from each household. This study was designed to have 80% power to detect an increase of contact coverage from 5 to 25%, assuming 10–20% of participants were probable cases, and with an intra-class correlation of 0.1 to account for village-level clustering. As relatively few probable cases were identified in the baseline round, the sample size was increased for the endline round to increase statistical power. Field workers orally administered a structured questionnaire to identify participants who had probable depression or AUD. The questionnaire contained sections on demographic characteristics, depression screening, depression symptoms in the past year, and alcohol use screening. A probable case of depression was defined as a participant who scored 10 or more on the 9 item Patient Health Questionnaire (PHQ-9) [20] or who responded affirmatively to a question about experiencing problems like those described in the PHQ-9 over another 2-week period in the past 1 year. The PHQ-9 has 9 items with a score ranging from 0 to 3 for each item. The PHQ-9 has been validated in Uganda and was found to perform well among patients with depression with area under the curve of 0.74–0.96 [21, 22].

A probable case of AUD was defined as a participant who scored 8 or more on the Alcohol Use Disorders Identification Test (AUDIT. The 10-item AUDIT was designed to identify hazardous drinkers, harmful drinkers and people with alcohol dependence. The tool was designed by World Health Organisation and can be used in both community and clinical settings [23]. The AUDIT has not been validated in Uganda but has been shown to have very good psychometric properties when validated in LMICs [24]. A meta-analysis of AUDIT validation studies to detect at-risk drinking reported an area under the curve of 0.92, albeit with high heterogeneity across studies [25].

Probable cases were asked about whether they had contacted a health care provider, classified as specialist (e.g. psychiatrist, psychiatric nurse, psychologist), complementary/traditional healer, or primary care provider (e.g. nurse, medical officer) to deal with their symptoms. The primary care provider category was the target of the PRIME MHCP. Epilepsy and psychosis were excluded from an analysis of change in contact coverage because their relative scarcity required a prohibitively large sample size to identify sufficient cases to achieve adequate statistical power.

#### Aim 2: facility level detection of depression and AUD

The facility detection study aimed to estimate the change in the proportion of adult attendees of primary health care clinics with probable depression and probable AUD who are detected by primary care practitioners. The baseline facility detection study (n = 1893) was conducted from June to November 2013 prior to training primary health care (PHC) workers in the WHO’s Mental Health Gap Action Program Intervention Guide (mhGAP-IG) [26]. Seventy two percent (72%) of primary health workers at health center levels III and IV (140 nurses and 32 physician assistants) were then trained for 5 days in mhGAP-IG. The training included the identification, diagnosis and treatment of selected mental disorders including depression, alcohol use disorder, psychosis and epilepsy. The trained health workers were provided with copies of the mhGAP intervention guidelines for reference and were supervised by a specialist mental health worker (Psychiatrist or psychiatric clinical officer) once every 3 months. Follow up cross-sectional surveys with independent samples were conducted 3 months (midline; n = 2094) and 6 months (endline; n = 1891) after training. This study was designed to have 80% power to detect an increase of detection from 5 to 30%, assuming 20% of participants were probable cases. This was based on the assumption that the treatment gap for common mental disorders in Kamuli district was estimated to be 95% given that it is a very low resourced district with hardly a mental health service. Hence only 5% were estimated to be getting any care at all. We aimed to detect at least 30% of the common mental disorders basing on Ustun et al. [27] study done in 15 primary care settings around the world. In this study a prevalence of 33 percent of common mental disorders was found in primary care.

Adult patients were recruited from the appointment registration desk at 12 primary health clinics (Level III and IV) and one primary care department in the district hospital. As consecutive adults registered, they were recruited to participate. Field workers verbally administered a structured pre-consultation questionnaire, which included sections on demographic characteristics, and screening for depression and AUD. As in aim 1 above, a probable case of depression had a PHQ-9 score of 10 or more and a probable case of AUD had an AUDIT score of 8 or more. The screened respondents then continued to their regular clinic consultation with a trained primary care worker who was blinded to the screening results. All probable cases were asked to return immediately after their clinical consultation with a primary care worker to be administered a structured post-consultation exit questionnaire about their consultation, and whether they received any relevant diagnoses (“Did the health worker give you a diagnosis of alcohol problems” and “Did the health worker give you a diagnosis of depression”). Field workers also checked the participants’ consultation notes for diagnoses. For the same reasons as in aim 1 above, epilepsy and psychosis were excluded from this aim.

#### Aim 3: treatment outcomes for depression, psychosis and epilepsy

The Treatment Cohort Studies aimed to estimate the change in symptom severity and functional impairment over 12 months among adults who were diagnosed with depression, epilepsy or psychosis by primary care clinicians and provided with treatment or referred for specialist care (for severe cases) in the implementation facilities. A 12 months follow-up period was chosen taking into account the longest duration of treatment of a single depression episode which is stipulated in the mhGAP intervention guidelines on which the health worker training was based [17]. Patients with psychosis and epilepsy who were recruited into the cohorts were clinically identified using the WHO mhGAP intervention guide algorithms. Patients with depression were screened using the PHQ-9 and diagnosis confirmed clinically by primary care worker using the mhGAP algorithms. The methods of this cohort study have previously been described [18]. Recruitment for the treatment cohorts took place between January and September 2015. AUD was excluded from aim 3 because too few probable cases were found presenting to clinics in the baseline facility detection study. The consort diagram in Additional file 1: Figure S2 demonstrates the flow of participants in the three study cohorts.

Eighteen months after implementation of the MHCP started, field workers were notified by clinicians when patients were diagnosed with depression, psychosis or epilepsy. On the same day, the field workers would contact and assess these patients for eligibility, obtain informed consent and conduct a baseline interview. Eligibility criteria comprised: age 18 years or over, receipt of the relevant clinical diagnosis by the primary care worker, residence in Kamuli district, and ability to understand the informed consent process. Field workers completed a midline interview either 3 months (depression) or 6 months (psychosis and epilepsy) after baseline, and an endline interview 12 months after baseline. Interview questionnaires contained sections on participants’ demographic characteristics, symptom severity, and impairment. Symptom severity was measured with the PHQ-9 for the depression cohort. At baseline the epilepsy cohort included patients who were both symptomatic, (i.e. had ≥ 1 seizure in the past 30 days) as well as those that were asymptomatic. Only those who were symptomatic were included in the analysis. Symptom severity for epilepsy was measured by the number of seizures in the last 30 days. For the psychosis cohort, the main outcome was functional impairment. No symptom severity measure was collected for the psychosis cohort since most of the participants had been on treatment for some time. Functional impairment was measured with the 12-item WHO Disability Assessment schedule 2.0 (WHODAS 2.0) for all three cohorts. The WHODAS 2.0 is a generic assessment tool for health and disability, which is appropriate for use across cultures in adult populations and has been found to be valid across 19 countries [28]. Each item is measured on a 5-point likert scale and item scores are summed to give the degree of functional limitation. Greater WHODAS scores indicate greater functional impairment [29].

A range of process indicators were identified through Theory of Change workshops during the MHCP development [30]. These indicators were measured though a range of other evaluation methods which have been described elsewhere [16]. These process indicators enable explanatory analysis of the outcomes presented here, and will be reported in a separate paper.

### Statistical analysis

First, we described the demographic and mental health characteristic of participants in each round of the community and facility studies, and for each treatment cohort at baseline, using means and standard deviations for continuous measures and counts and percentages for categorical variables.

#### Aim 1: contact coverage for depression and AUD

We tabulated the proportion of adults with probable depression in the population who contacted a primary care provider at each round. For the follow up round, we estimated the change in proportion of probable cases that contacted a primary care provider and the 95% confidence interval using binomial regression. Due to imbalances of participant characteristics across survey rounds we included age, sex and screening score in the binomial regression model as confounders and reported the adjusted figures when those models converged. When it was not possible to estimate the change from baseline using binomial regression (i.e. because the baseline value was 0.0), we estimated a one-sample proportion with 95% CI and tested it against a hypothesized value of zero. Then, when sufficient follow up data were available, we assessed inequity of provider contact by stratifying by sex and by educational attainment, and tested for associations using a Chi-square test. A Chi square P < 0.10 was considered suggestive of inequity. We repeated the above analyses for adults with probable AUD, and then for both disorders by expanding the outcome to include any health provider (i.e. specialist, primary care, complementary/traditional). Counts were reported as observed, while proportions, differences, 95% CI and P-values were design-adjusted for the population-based survey design.

#### Aim 2: facility level detection of depression and AUD

For each round of the facility study we tabulated the proportion of PHQ-9 positive adults in health facilities who reported or whose consultation notes indicated that they had been clinically detected for depression. The same applied for AUDIT-positive adults diagnosed as AUD. Compared to the baseline round, for each disorder we estimated the change in detection and 95% confidence interval using binomial regression. Though there were imbalances of participant characteristics across study round, binomial regression models with these characteristics included as confounders did not converge, and so we reported results from univariable models. When it was not possible to estimate the change from baseline using binomial regression (i.e. because the baseline value was 0.0), we estimated a one-sample proportion with 95% CI and tested it against a hypothesized value of zero. Next, at each follow-up round, when sufficient data were available, we tested for inequity of detection by sex and by educational attainment using Fisher’s Exact test. A Fisher’s Exact P < 0.10 was considered suggestive of inequity.

#### Aim 3: treatment outcomes for depression, psychosis and epilepsy

For the depression cohort, we calculated the mean PHQ-9 score at each interview round. We estimated the change in symptom severity score at each follow up round in comparison to the baseline and its 95% confidence interval using negative binomial regression. For the epilepsy cohort, the number of seizures in the past 30 days was the outcome of interest, and, due to the presence of outliers, we used the Wilcoxon rank-sum test to compare each follow up visit against the baseline. For each cohort we calculated the mean WHODAS score at each follow up round and used negative binomial regression to estimate the change in mean score since the baseline. For the psychosis cohort, the main outcome was functional impairment. No symptom severity measure was collected for this cohort.

Next, we considered inequity of changes in symptom severity and change in impairment across gender and education attainment. For all cohorts, inequity of change in impairment was conducted by adding interaction terms for sex and for educational attainment to the regression models. This was also done for change in symptom severity for the depression cohort. Each set of interaction terms were tested using the Wald test. For the epilepsy cohort participants, the median change in number of seizures from baseline to each follow-up was compared across gender using the Wilcoxon rank sum test, and across education using the Kruskal–Wallis test. Inequity test P-values < 0.10 were considered suggestive of inequity.

### Ethics

All participants provided written informed consent by signing a study consent form. For those who were illiterate, the contents of the consent form were read to them in local language by a research assistant before they appended a thumb print. All patients who were found to be at risk of suicide were referred to the mental health specialist at the district hospital. All adverse events during the cohort study were documented in a register of adverse events.

Ethical approval of the protocols for all the studies was obtained from Makerere University (Kampala, Uganda), the Uganda National Council of Science and Technology (Kampala, Uganda), the University of Cape Town (South Africa), and the World Health Organization (Geneva, Switzerland). Institutional approval was also obtained from the district administration for all the studies.

## Results

The demographic and mental health characteristics of participants are presented in Table 2.

**Table 2:** Demographic and mental health characteristics of Community Study, Facility Study and Treatment Cohort participants in PRIME evaluations, Kamuli District, Uganda, 2013–2017

Characteristic	Community study^a^		Facility study			Treatment cohort studies		
Level	Baseline (n = 1290)	Endline (n = 3481)	Baseline (n = 1893)	Midline (n = 2049)	Endline (n = 1891)	Depression (n = 64)	Psychosis (n = 51)	Epilepsy (n = 117)
Age (years), %								
16–30	494 (38.2)	1190 (32.7)	1035 (54.7)	678 (35.8)	180 (9.5)	18 (28.1)	26 (51.0)	83 (70.9)
31–49	555 (44.2)	1424 (42.3)	991 (48.4)	799 (39.0)	259 (12.6)	34 (53.1)	23 (45.1)	31 (26.5)
≥ 50	240 (17.6)	867 (25.0)	862 (45.6)	732 (38.7)	297 (15.7)	12 (18.8)	2 (3.9)	3 (2.6)
Female sex, %	867 (66.4)	2668 (74.7)	1500 (79.2)	1618 (78.9)	1541 (81.4)	48 (75.0)	23 (45.1)	56 (47.9)
Marital status								
No partner						24 (37.5)	36 (70.6)	103 (88.0)
Has a partner						40 (62.5)	15 (29.4)	14 (12.0)
Highest education^b^, %								
Less than primary	239 (16.9)	663 (18.2)	174 (9.2)	348 (17.0)	843 (44.6)	15 (23.4)	1 (2.0)	45 (24.9)
Primary	685 (52.6)	1960 (55.8)	1113 (58.8)	1177 (57.4)	648 (34.2)	–	–	–
Secondary	322 (26.7)	759 (23.0)	485 (25.6)	473 (23.1)	362 (19.1)	36 (56.3)	21 (41.2)	115 (63.5)
Tertiary	44 (3.7)	99 (3.0)	121 (6.4)	52 (2.5)	39 (2.1)	13 (20.3)	29 (56.9)	21 (11.6)
PHQ-9 score	3.9 (2.4)	2.2 (3.5)	3.0 (2.9)	3.6 (3.5)	3.3 (3.3)	16.3 (4.6)	9.5 (6.5)	7.6 (5.6)
PHQ9 positive, %	85 (6.7)	302 (8.6)	80 (4.2)	158 (7.7)	111 (5.9)	61 (95.3)	23 (45.1)	46 (39.3)
Recent depression, %	316 (24.9)	380 (10.7)	159 (8.4)		314 (16.6)			
AUDIT score	0.6 (1.6)	0.6 (2.7)	0.6 (1.8)	0.5 (1.9)	0.6 (2.5)			
AUDIT positive, %	25 (2.3)	63 (1.8)	23 (1.2)	38 (1.8)	48 (2.5)			
Number of seizures in past 30 days (median, IQR)								3 (1–5)

Mean (SD) for continuous variable, count (%) for categorical variables

^a^Counts are reported as observed, while proportions, 95% CI and P-values are design-adjusted for the population-based survey design

^b^In the cohort study, response options ‘Uneducated’ and ‘Non formal’ were combined into ‘Less than primary school’

### Aim 1: contact coverage for depression and AUD

The baseline community study round (n = 1290) was conducted from May to June 2013, and the follow up (n = 3481) from August 2016 to March 2017. The response rate was 100% for both the baseline and endline survey. Of the baseline participants 325 (25.4%) were either PHQ-9 positive (n = 85, 6.7%) or had recent history of depression symptoms (n = 316, 24.9%), and so were considered to be probable cases of depression. Twenty five (2.3%) baseline participants were AUDIT positive and were probable cases of AUD.

Using community survey data, the proportion of probable cases who contacted a health provider at each round are reported in Table 3. In the baseline round, of the 325 probable cases of depression, 48 (16.5%) contacted a primary care provider about their symptoms. The corresponding proportion in the follow up round was 19.4%, which was a non-significant increase of 4.1% (95% CI − 1.8, 10.1). For probable cases of AUD, contact with a primary care provider increased by 1.3% (95% CI − 1.3 to 3.9). The differences for contact with any health provider (i.e. primary, specialist, complementary/traditional) among people with probable depression or probable AUD were also not statistically significant.

**Table 3:** Contact with a health care provider for adults with probable depression or alcohol use disorder in Kamuli District, Uganda, 2013–2017

	Baseline (n = 1290)	Endline (n = 3481)	Contact difference (95% CI)^a^	P-value^a^
Depression				
Probable case (%)	325/1290 (25.4)	452/3481 (12.9)		
Contact with primary care provider	48/325 (16.5)	94/452 (19.4)	+ 4.1 (− 1.8, 10.1)^c^	0.173^c^
Contact with any health provider	67/325 (23.1)	101/452 (20.9)	− 0.1 (− 8.5, 6.8)^c^	0.825^c^
Alcohol use disorder				
Probable case (%)	25/1290 (2.3)	63/3481 (1.8)		
Contact with primary care provider	0/25 (0.0)	1/63 (1.3)	+ 1.3 (− 1.3, 3.9)^b^	0.317^b^
Contact with any health provider	1/25 (2.7)	6/62 (9.5)	+ 6.8 (− 1.7, 15.4)	0.117

Counts are reported as observed, while proportions, differences, 95% CI and P-values are design-adjusted for the population-based survey design

^a^Estimated with univariable binomial regression, unless otherwise indicated

^b^Estimated with one-sample test of proportion vs. H_0_ value of 0%

^c^Adjusted for age, sex and PHQ9 score

There was evidence (Chi-square P = 0.046) of inequity of contact with a primary provider by gender in favour of women, in that more women with probable depression (80 of 351; 21.3%) contacted a primary provider in the follow up round, compared to 14 of 101 (13.6%) men with probable depression. There was no evidence of inequity (Chi-square P = 0.780) of contact for probable depression by education level, and there was insufficient data to assess inequity for AUD treatment contact (Additional file 1: Table S1).

### Aim 2: facility level detection of depression and AUD

The baseline facility study (n = 1893) was conducted from June to November 2013, with follow up rounds conducted from Aug to Oct 2014 (n = 2094) and Feb to April 2016 (n = 1891). All participants provided informed consent. Of the baseline participants, 80 (4.2%) were PHQ-9 positive and 23 (1.2%) were AUDIT positive.

The proportions of probable cases who were clinically detected at each round are reported in Table 4. In the baseline round, 80 of 1893 (4.2%) participants were probable cases of depression, 48 of 80 were re-contacted after their clinical consultation and 2/48 (4.2%) had been appropriately diagnosed by primary health care staff. In the midline round, detection was 12.7%, an increase of 8.6% (95% CI 0.8, 16.4; P = 0.031). At endline, the increase in detection from baseline was 0.6% (95% CI − 6.3, 7.7; P = 0.848). No probable cases of AUD were detected at baseline, precluding estimation of change using binomial regression. Using a one-sample test of proportion again a hypothesized value of 0.0, 4/32 cases were detected at midline (12.5%, 95% CI 0.7, 24.3; P = 0.038) and 2/38 cases (5.3%, 95% CI − 0.2, 12.6; P = 0.155) at endline.

**Table 4:** Clinical detection of depression and of alcohol use disorder among adult outpatients in PRIME implementation clinics in Kamuli District, Uganda, 2013–2017

	Baseline (n = 1893)	Midline (3 months) (n = 2050)	Endline (6 months) (n = 1892)
Depression			
Screen positive, n (%)	80/1893 (4.2)	158/2050 (7.7)	111/1892 (5.9)
Exit data available, n (%)	48/80 (60.0)	149/158 (94.3)	103/111 (92.8)
Detected, n (%)	2/48 (4.2)	19/149 (12.7)	5/103 (4.8)
Change vs baseline, % (95% CI)^a^		+ 8.6 (0.8, 16.4)	+ 0.6 (− 6.3, 7.7)
P-value^a^		0.031	0.848
Alcohol use disorder			
Screen positive, n (%)	23/1893 (1.2)	38/2050 (1.8)	48/1892 (2.5)
Exit data available, n (%)	18/23 (78.3)	32/38 (84.2)	38/48 (79.2)
Detected, n (%)	0/18 (0.0)	4/32 (12.5)	2/38 (5.3)
Detected, % (95% CI)^b^		12.5 (0.7, 24.3)	5.3 (− 0.2, 12.6)
P-value^b^		0.038	0.155

^a^Calculated with binomial regression

^b^Calculated with one-sample test of proportion vs. 0.0

There was evidence of an inequitable distribution of clinical detection of depression by education status at the midline round (Fisher’s P = 0.028), in that 12 of 54 (22.2%) of those with less than primary education were detected, compared to 5 of 77 (6.5%) with primary education and 2 of 18 (11.1%) with tertiary education or more. At endline again there was evidence of inequity (Fisher’s P = 0.053) when the corresponding percentages were 2.3, 2.4 and 17.6% respectively. There was no evidence of inequity (Fisher’s P > 0.10) for detection of depression by gender, or for detection of AUD by gender or by education (Additional file 1: Table S2).

### Aim 3: treatment outcomes for depression, psychosis and epilepsy

Recruitment for the Treatment Cohorts took place between January and September 2015. Additional file 1: Figure S2 shows the consort diagram for the cohort studies. Of the 80 patients diagnosed with depression 64 completed the baseline interview, of whom 3 were lost to follow up after 3 months and 7 (10.9%) were lost to follow up after 12 months. Fifty one patients were diagnosed with psychosis and completed the baseline interview. Of these 4 (7.8%) were lost to follow up after 6 months and 8 (15.7%) after 12 months. A total of 117 patients were diagnosed with epilepsy and were symptomatic at baseline. Of these, 3 (2.6%) were lost to follow up after 6 months and 10 (8.5%) after 12 months.

Using treatment cohort data, the levels and changes in mean symptom severity score at each visit are reported in Table 5. At baseline, the participants in the depression cohort had a mean PHQ-9 score of 16.3 (SD 4.6), which decreased by 7.9 points (95% CI − 12.6, − 3.2) to 8.4 (SD 6.3) after 3 months. The mean score after 12 months was 6.4 (SD 5.6), which was a reduction of 9.9 points (95% CI − 14.4, − 5.4) from baseline. Epilepsy patients had a median of 3 seizures (IQR 1–5) in the 30 days prior to baseline, which was lower at 6 months (median 1, IQR 0–4, P < 0.05) and at 12 months (median 2, IQR 0–4, P < 0.05). As seen in Additional file 1: Table S3, there was no evidence of inequity for symptom severity outcomes by gender or by education in any cohort.

**Table 5:** Change in symptom severity and impairment score for depression, epilepsy and psychosis patients at PRIME implementation clinics in Kamuli District, Uganda, 2015–2017

Disorder	Measurement	Baseline score	Midline^a^ score	Endline score	Midline^a^ difference in score vs. baseline (95% CI)	Endline difference in score vs. baseline (95% CI)
Outcome						
Depression						
Symptom severity	Mean PHQ-9 (SD)	16.3 (4.6)	8.4* (6.3)	6.4* (5.6)	− 7.9 (− 12.6, − 3.2)	− 9.9 (− 14.4, − 5.4)
Impairment	Mean WHODAS (SD)	48.4 (18.0)	26.7* (22.1)	22.6* (22.3)	− 21.7 (− 35.4, − 7.9)	− 25.8 (− 39.2, − 12.4)
Psychosis						
Impairment	Mean WHODAS (SD)	34.9 (27.2)	14.2* (18.8)	15.7* (20.9)	− 20.7 (− 31.3, − 10.1)	− 19.2 (− 30.1, − 8.4)
Epilepsy						
Symptom severity	Median # seizures in past 30 days (IQR)	3 (1–5)	1** (0–4)	2** (0–4)		
Impairment	Mean WHODAS (SD)	35.9 (23.6)	26.9* (29.0)	23.5* (27.9)	− 9.1 (− 17.4, − 0.8)	− 12.4 (− 20.5, − 4.4)

* Negative binomial regression P < 0.05 for difference of score vs. baseline

** Wilcoxon Sign-rank P < 0.05 for difference of score vs. baseline

^a^3 months for depression, 6 months for psychosis and epilepsy

The levels and changes in mean functional impairment score at each visit are also reported in Table 5. At baseline, participants in the depression cohort had a mean WHODAS score of 48.4 (SD 18.0), which decreased by 21.7 points (95% CI − 35.4, − 7.9) to 14.2 (SD 18.8) after 3 months. The mean score after 12 months was 22.6 (SD 22.3), which was a reduction of 25.8 points (95% CI − 39.2, − 12.4) from baseline. At baseline, the psychosis cohort had a mean WHODAS score of 34.9, which decreased by 20.7 points (95% − 31.3, − 10.1) after 6 months (p < 0.05), and by 19.2 points (95% CI − 30.1, − 8.4) after 12 months from baseline (p < 0.05). Patients in the epilepsy cohort had a mean WHODAS score of 17.4 at baseline, and there was no evidence of a change after 6 or 12 months follow up. There was no evidence of inequity for impairment outcomes by gender or by education in any cohort (Additional file 1: Table S3).

## Discussion

This study presents findings of impacts at multiple levels following implementation of the Kamuli District Mental Health Care plan in Uganda. There was negligible change in population-level contact coverage for depression and AUD. For adults who did attend primary care facilities, there was an immediate positive effect of the MHCP on clinical detection, although this was not sustained over time. Those who were treated in primary care experienced significant reductions in symptom severity and functional impairment. In short, the study found that it is possible to integrate mental health into primary care in a manner that improves detection of common mental health problems, and improves clinical and functioning outcomes, although challenges remain in creating demand for services among probable cases and in sustaining these health system improvements over time.

### Aim 1: contact coverage

Closing the large treatment gap for mental disorders in LMICs necessitates that mental health care is integrated into primary care in order to increase coverage, and for people in need to make contact with a health provider. However, studies that evaluate contact coverage are few globally and nearly non-existent in LMICs. Our study provides a methodological framework for such an evaluation to be conducted in other LMICs. The impact of implementing the MHCP on population-level contact coverage was small for depression and AUD in this study. Although this measure of contact coverage assumes that everyone who screens positive for a mental disorder is in need of treatment, the decision to seek treatment may be influenced by a range of factors including severity of illness, suitability of treatment provided, other sources of support and preferences of individual patients which may be determined by social cultural influences [31]. In Kamuli district, the MHCP included community interventions to raise awareness through the local media about mental disorders and where to seek help. However, such interventions may have been insufficient to affect coverage because of the large network of traditional healers from whom many people ordinarily prefer to seek help when in distress. Social desirability bias may have been at play in which case respondents give answers to interview questions based on what they think is desired or acceptable by the interviewer or by society. In addition, in Uganda, alcohol use is often not deemed an issue that requires medical treatment, which may partly explain the low contact coverage for AUD. Geographical distance may also have hindered access to a PHC worker. In Kamuli health centers are within a 5–10 km radius which is quite a distance where most people do not own vehicles. These factors influence help seeking. There was need to address these issues at the community level. However, these personal and structural factors were beyond the capacity and scope of this study to influence. Lastly, a 36 months implementation period may have been too short for help seeking behavior to change. Methodological limitations may also have led to a change in coverage that was lower than expected. We depended on participants recalling whether they attended with a health provider for particular mental health conditions which could have introduced recall bias.

### Aim 2: clinical detection

Clinical detection of mental disorders for those who have made contact with a health provider is a necessary next step in closing the treatment gap. However, detection of mental disorders in primary care is low in LMICs and few detection studies are available to inform health system planners in these countries. Our study supports this with a detection rate at baseline of 4.2% and 0% for depression and AUD respectively. Studies in high income countries suggest international variability in detection rates by primary health care workers compared to case finding tools ranging from 23% in Korea [32] to 45% in the United Kingdom to 64% in Italy [33]. The heterogeneity observed in all these studies should be the subject of future research, to identify the factors associated with higher or lower detection and to guide intervention planning in LMIC settings.

In-service brief training of primary care workers has been found to improve the rate of detection of mental disorders in primary care and to impact treatment outcomes [5]. Our study attests to this at least in the first 3 months from baseline. However, the gains in clinical detection were not sustained possibly due to decay of knowledge and skills over time, a fact highlighted in previous studies [34]. Gilbody et al. [35] in their study suggested that complex integrated quality improvement strategies that involve health worker education as well as patient level interventions such as patient education, nurse case management, and enhanced support from specialist services may be more effective in sustaining patient outcomes over a 12 months period. In Kamuli, although prior training of primary care workers and 3 monthly supervision by a specialist nurse or clinician were done, detection remained low. Future research is needed to determine what intensity of training and supervision is sufficient and what other factors might affect clinicians’ ability to sustain a higher level of detection.

Various factors may have determine the low detection of depression and AUD in primary care in Kamuli district. For depression, factors highlighted by Manson and Kirmayer [36, 37] could have been at play. These include possible differences in symptom presentation as well as in cultural concepts and idioms of depression across cultures, and the different conceptualization of depression by PHC workers compared to specialists. In a study done among the Baganda in central Ugandan who share cultural norms with the population in Kamuli, depression was found to be conceptualized as a disease of cognition (too much thoughts) rather than emotion (sadness) [38] and yet the later is what defines depression in most diagnostic guidelines as well as screening tools internationally. This calls into question the validity of the PHQ-9 as a case finding tool for depression in this study population. This is particularly important in LMICs where most studies use screening tools developed in high income settings. Despite the PHQ-9 having been successfully validated in several populations in LMIC (including some populations in Uganda) it is possible that when the PHQ-9 was applied to the Kamuli population, it did not identify the same individuals with depression as the PHC worker, leading to a low PHC worker detection rate compared to the case finding tool. This highlights the need for population specific validation of such tools.

Detection of AUD could have been limited by the fact that majority of participants in the primary care study clinics in Kamuli were female. In Uganda, it is mostly women who attend the PHC clinics either for themselves (for reproductive health reasons) or for their sick children. This may have introduced selection bias in the study population as fewer men, who are much more likely to suffer from AUD [39] attend health clinics. In addition, more than half of the men who screened positive for AUD in Kamuli in a previous study reported not seeking any help because they did not think AUD was a health condition which required medical treatment [40].

### Aim 3: symptom severity and functional impairment

Numerous randomized controlled trials have highlighted the effectiveness of integrated task shifted evidence-based interventions on improving patient outcomes [34]. These studies hold promise for many LMICs such as Uganda where few specialist mental health workers are available. Our study demonstrated that in a real-world complex evaluation, mental health treatment provided by trained and supervised primary health care workers can lead to improvement in symptom severity and functioning of patients with depression and epilepsy, and in functioning in patients with psychosis.

### Equity

Equity in health care has been variously defined by different disciplines and perspectives. Many of these perspectives agree on a few common principles of equity. These include (i) equal access for those with equal need; (ii) equal utilization for those with equal need; and (iii) equal or equitable health outcomes Of these, equal access for those with equal need is the most widely used. Access may be influenced by demand side issues including literacy, knowledge and information availability, geographical differences, income disparities, gender, cultural beliefs and culturally determined health seeking behavior and individual preferences regarding whether and where to seek care. In this study, since there was more homogeneity with regard to income status, geographical location and culture, we aimed to examine inequity with regard to contact with a primary provider or treatment outcomes related to gender or education status only. There was no evidence of such inequity for women or those with no or low education attainment, which was an important finding. However, for the men it was evident that the primary care setting may not be the best place for treating their mental disorders as they rarely use the services. It may also be due to their belief that conditions such as AUD are not health problems needing treatment at a health facility [40]. Another possible cause of inequity towards men is stigma towards them seeking help for conditions like depression or AUD and a belief that they may be showing weakness. These issues will be addressed in a future secondary analysis. Other strategies may be needed to increase access to care among men.

### Strengths and limitations

The strengths of this study included the fact that this was a real-world complex evaluation. This makes it possible to scale up to other real-world settings. The theory of change framework used to develop and evaluate the district mental health care plan ensured participation of all stakeholders and that the intervention was appropriate and acceptable to the stakeholders. The study tools used have been widely used and some previously validated in Uganda and other LMICs which made them appropriate for use in this study. Even without a comparison group, the change in detection of depression between baseline and midline was likely due to the district MHCP since there were no other such interventions in the district to influence contact coverage, clinical detection or clinical outcomes for people with mental, neurological or substance use disorders.

Nonetheless there were some limitations, the strengths notwithstanding. First, in the community and facility studies, the proportion of people screening positive for AUD was unexpectedly low, leading to low power to detect effects for changes in contact coverage and in clinical detection of AUD. Second, the reliance on repeated measures and the lack of a comparison group could have introduced a regression to the mean effect. However, we considered it ethically inappropriate to recruit people with a mental disorder into a non-intervention control arm and not provide care. We considered that in a setting where there is nearly no care at all, a before-and-after study may suffice since the baseline round may serve as the no treatment comparison. Third, the majority of our respondents were female. Although this reflects the usual population at the primary centers in Uganda, it clearly didn’t adequately address the male population and the results can therefore not be generalized to the males in the study setting. Fourth, for the depression cohort, improvement due to normal disease progression could not be ruled out. Fifth, we were not able to assess changes in treatment coverage or detection for psychosis and epilepsy, due to the large sample size that would have been required to obtain the denominator for treatment coverage, a sample that was beyond the resources available for the study.

## Policy implications

The PRIME Kamuli district MHCP plan provides a scalable model for integrating mental health into primary care in low-resource settings in Africa. Despite challenges with demonstrating an increase in contact coverage, there is evidence that integration of mental health care into primary care is a worthwhile endeavor. We further demonstrated that treatment of mental disorders by trained non-specialist primary health care workers can lead to significant improvements in clinical symptoms and functional impairment. The lesson therefore for health planners is that it is possible to improve access to effective mental health care with the available resources at primary health care level.

However, deliberate efforts need to be made to access men who need care for depression or AUD as the primary care clinics may not be an appropriate place for them to be targeted. Further work is needed to elucidate the nature and quantity of interventions sufficient to increase contact coverage significantly and to sustain the initial improvements in detection. Areas for further research include methods of awareness raising to improve help seeking; nature and amount of supportive supervision to trained primary health workers and nature, frequency and duration of refresher training.

The key development objective of “leave no one behind”, enshrined in the Sustainable Development Goals is an important one in the field of global mental health. In this study we endeavored to address inequity in mental health care provision towards vulnerable groups such as women and the uneducated. This was achieved by PRIME Uganda with no evidence of inequity by gender or education levels in the clinical or functioning outcomes. Even though more thought needs to be put into how men with AUD can be better reached for care in this low resourced setting, this study, nonetheless, creates a case for greater investment in the integration of mental health into primary care.

## Conclusion

We have demonstrated a mixed methods approach to implementing and evaluating a complex intervention in the real world which could be replicated. There was a positive impact on the clinical and functional outcomes of people with depression, psychosis, and epilepsy when the district MHCP was implemented by trained non-specialist primary health care workers. The improvement in detection of depression and AUD at 3 months after training of primary health care workers was not sustained over 12 months. The adult males were not adequately targeted by a mental health intervention in the studied primary care setting. Future research needs to address the factors that influence a decision to contact a health care provider by people with mental disorders especially the men and the nature and quantity of interventions needed to improve help seeking and therefore contact coverage. There is also need for further research into how improvement in detection rates in primary care can be sustained.

## Supplementary information

**Additional file 1: Figure S1.** Implementation and Evaluation timeline for mental health care plan in Kamuli District, Uganda. **Figure S2.** Consort diagram – treatment cohort studies for mental health patients of primary health care centers in Kamuli District, Uganda. **Table S1.** Inequality of contact with a primary health care provider for adults with probable depression in Kamuli District, Uganda, 2017. **Table S2.** Inequality of clinical detection of depression and of alcohol use disorder among adult outpatients in PRIME implementation clinics in Kamuli, Uganda, 2014 & 2017. **Table S3**. Inequity of change in symptom severity and impairment score for depression, epilepsy and psychosis patients at PRIME implementation clinics in Kamuli, Uganda, 2015-2017.

## Data Availability

“The data will be made available on 31^st^ October 2019 at ZivaHub (https://zivahub.uct.ac.za/) at University of Cape Town. Data will be availed upon reasonable request, by completing an ‘Expression of Interest form’ available here: http://www.prime.uct.ac.za/contact-prime. The data collection instruments used for this study are also in the process of being made available to the public on the PRIME website (http://www.prime.uct.ac.za).”

## References

[1] Vigo D, Thornicroft G, Atun R (2016). Estimating the true global burden of mental illness. Lancet Psychiatry..

[2] Mathers CD, Loncar D (2006). Projections of global mortality and burden of disease from 2002 to 2030. PLoS Med.

[3] Ovuga E, Boardman J, Wasserman D (2005). The prevalence of depression in two districts of Uganda. Soc Psychiatry Psychiatr Epidemiol.

[4] Demyttenaere K, Bruffaerts R, Posada-Villa J, Gasquet I, Kovess V, Lepine J, Angermeyer MC, Bernert S, Morosini P, Polidori G, Kikkawa T (2004). Prevalence, severity, and unmet need for treatment of mental disorders in the World Health Organization World Mental Health Surveys. JAMA.

[5] Kroenke K, Taylor-Vaisey A, Dietrich AJ, Oxman TE (2000). Interventions to improve provider diagnosis and treatment of mental disorders in primary care: a critical review of the literature. Psychosomatics..

[6] Funk M (2008). Integrating mental health into primary care: a global perspective.

[7] Patel V, Araya R, Chatterjee S, Chisholm D, Cohen A, De Silva M, Hosman C, McGuire H, Rojas G, van Ommeren M (2007). Treatment and prevention of mental disorders in low-income and middle-income countries. Lancet..

[8] Gilbody S, Whitty P, Grimshaw J, Thomas R (2003). Educational and organizational interventions to improve the management of depression in primary care: a systematic review. JAMA.

[9] Lund C, Tomlinson M, De Silva M, Fekadu A, Shidhaye R, Jordans M, Petersen I, Bhana A, Kigozi F, Prince M, Thornicroft G (2012). PRIME: a program to reduce the treatment gap for mental disorders in five low-and middle-income countries. PLoS Med.

[10] Hanlon C, Luitel NP, Kathree T, Murhar V, Shrivasta S, Medhin G, Ssebunnya J, Fekadu A, Shidhaye R, Petersen I, Jordans M (2014). Challenges and opportunities for implementing integrated mental health care: a district level situation analysis from five low-and middle-income countries. PLoS ONE.

[11] Uganda Bureau of Statistics (UBOS) and ICF. Uganda Demographic and Health Survey 2016. Kampala: UBOS and ICF; 2018. https://dhsprogram.com/pubs/pdf/FR333/FR333.pdf. Accessed 4 Jan 2019.

[12] Uganda Hospital and Health Centre IV Census Survey; 2014. https://www.who.int/healthinfo/systems/SARA_H_UGA_Results_2014.pdf. Accessed 4 Jan 2019.

[13] Kirkpatrick DL (1959). Techniques for evaluating training programs. J Am Soc for Train Dev..

[14] De Silva MJ, Breuer E, Lee L, Asher L, Chowdhary N, Lund C, Patel V (2014). Theory of Change: a theory-driven approach to enhance the Medical Research Council’s framework for complex interventions. Trials..

[15] Kigozi FN, Kizza D, Nakku J, Ssebunnya J, Ndyanabangi S, Nakiganda B, Lund C, Patel V (2016). Development of a district mental healthcare plan in Uganda. Br J Psychiatry..

[16] De Silva MJ, Rathod SD, Hanlon C, Breuer E, Chisholm D, Fekadu A, Jordans M, Kigozi F, Petersen I, Shidhaye R, Medhin G (2016). Evaluation of district mental healthcare plans: the PRIME consortium methodology. Br J Psychiatry.

[17] World Health Organization. mhGAP intervention guide for mental, neurological and substance use disorders in non-specialized health settings: mental health Gap Action Programme mhGAP, version 2.0. Geneva: World Health Organization; 2016. http://www.who.int/iris/handle/10665/25023. Accessed 5 Jan 2019. 27786430

[18] Rathod SD, De Silva MJ, Ssebunnya J, Breuer E, Murhar V, Luitel NP, Medhin G, Kigozi F, Shidhaye R, Fekadu A, Jordans M (2016). Treatment contact coverage for probable depressive and probable alcohol use disorders in four low-and middle-income country districts: the PRIME cross-sectional community surveys. PLoS ONE.

[19] Baron EC, Rathod SD, Hanlon C, Prince M, Fedaku A, Kigozi F, Jordans M, Luitel NP, Medhin G, Murhar V, Nakku J (2018). Impact of district mental health care plans on symptom severity and functioning of patients with priority mental health conditions: the Programme for Improving Mental Health Care (PRIME) cohort protocol. BMC Psychiatry..

[20] Rathod SD, Roberts T, Medhin G, Murhar V, Samudre S, Luitel NP, Selohilwe O, Ssebunnya J, Jordans MJ, Bhana A, Petersen I, Kigozi F, Nakku J, Lund C, Fekadu A, Shindhaye R (2018). Detection and treatment initiation for depression and alcohol use disorders: facility-based cross-sectional studies in five low-income and middle-income country districts. BMJ open..

[21] Kroenke K, Spitzer RL, Williams JB (2001). The PHQ-9: validity of a brief depression severity measure. J Gen Intern Med.

[22] Akena D, Joska J, Obuku EA, Stein DJ (2013). Sensitivity and specificity of clinician administered screening instruments in detecting depression among HIV-positive individuals in Uganda. AIDS Care..

[23] Nakku JEM, Rathod SD, Kizza D, Breuer E, Mutyaba K, Baron EC, Ssebunnya J, Kigozi F (2016). Validity and diagnostic accuracy of the Luganda version of the 9-Item and 2-Item Patient Health Questionnaire for detecting major depressive disorder in rural Uganda. Glob Mental Health.

[24] Saunders JB, Aasland OG, Babor TF, De la Fuente JR, Grant M (1993). Development of the alcohol use disorders identification test (AUDIT): WHO collaborative project on early detection of persons with harmful alcohol consumption‐II. Addiction.

[25] Cremonte M, Ledesma RD, Cherpitel CJ, Borges G (2010). Psychometric properties of alcohol screening tests in the emergency department in Argentina, Mexico and the United States. Addict Behav.

[26] Berner MM, Kriston L, Bentele M, Härter M (2007). The alcohol use disorders identification test for detecting at-risk drinking: a systematic review and meta-analysis. J Stud Alcohol Drugs.

[27] Üstün TB, Sartorius N (1995). Mental illness in general health care: an international study.

[28] Üstün TB (2010). Measuring health and disability: Manual for WHO disability assessment schedule WHODAS 2.0.

[29] Andrews G, Kemp A, Sunderland M, Von Korff M, Ustun TB (2009). Normative data for the 12 item WHO Disability Assessment Schedule 2.0. PLoS ONE..

[30] Breuer E, De Silva MJ, Shidhaye R, Petersen I, Nakku J, Jordans MJ, Fekadu A, Lund C (2016). Planning and evaluating mental health services in low-and middle-income countries using theory of change. Br J Psychiatry..

[31] De Silva MJ, Lee L, Fuhr DC, Rathod S, Chisholm D, Schellenberg J, Patel V (2014). Estimating the coverage of mental health programs: a systematic review. Int J Epidemiol.

[32] Chin WY, Chan KT, Lam CL, Wong SY, Fong DY, Lo YY, Lam TP, Chiu BC (2014). Detection and management of depression in adult primary care patients in Hong Kong: a cross-sectional survey conducted by a primary care practice-based research network. BMC Family Pract.

[33] Mitchell AJ, Rao S, Vaze A (2011). International comparison of clinicians’ ability to identify depression in primary care: meta-analysis and meta-regression of predictors. Br J Gen Pract.

[34] Gureje O, Abdulmalik J, Kola L, Musa E, Yasamy MT, Adebayo K (2015). Integrating mental health into primary care in Nigeria: report of a demonstration project using the mental health gap action program intervention guide. BMC Health Serv Res.

[35] Gilbody SM, Whitty PM, Grimshaw JM, Thomas RE (2003). Improving the detection and management of depression in primary care. BMJ Qual Saf.

[36] Manson SM (1995). Culture and major depression: current challenges in the diagnosis of mood disorders. Psychiatric Clin.

[37] Kirmayer LJ (2001). Cultural variations in the clinical presentation of depression and anxiety: implications for diagnosis and treatment. J Clin Psychiatry.

[38] Okello ES, Ekblad S (2006). Lay concepts of depression among the Baganda of Uganda: a pilot study. Trans Psychiatry..

[39] Keyes KM, Grant BF, Hasin DS (2008). Evidence for a closing gender gap in alcohol use, abuse, and dependence in the United States population. Drug Alcohol Depend.

[40] Nalwadda O, Rathod SD, Nakku J, Lund C, Prince M, Kigozi F (2018). Alcohol use in a rural district in Uganda: findings from community-based and facility-based cross-sectional studies. Int J Mental Health Syst.

