# Supplementary material for: Evaluation of the impacts of a district-level mental health care plan on contact coverage, detection and individual outcomes in rural Uganda: a mixed methods approach
Source: Int J Ment Health Syst. 2019 Sep 30;13:63. doi: 10.1186/s13033-019-0319-2 (PMC6767634; doi:10.1186/s13033-019-0319-2)
Supplement: Supplementary file 1 — Additional file 1: Figure S1. Implementation and Evaluation timeline for mental health care plan in Kamuli District, Uganda. Figure S2. Consort diagram – treatment cohort studies for mental health patients of primary health care centers in Kamuli District, Uganda. Table S1. Inequality of contact with a primary health care provider for adults with probable depression in Kamuli District, Uganda, 2017. Table S2. Inequality of clinical detection of depression and of alcohol use disorder among adult outpatients in PRIME implementation clinics in Kamuli, Uganda, 2014 & 2017. Table S3. Inequity of change in symptom severity and impairment score for depression, epilepsy and psychosis patients at PRIME implementation clinics in Kamuli, Uganda, 2015-2017. [file 13033_2019_319_MOESM1_ESM.docx]

**Figure S1: Implementation and Evaluation timeline for mental health care plan in Kamuli District, Uganda**

| Year | Quarter | Evaluation | | | | |  | Implementation | | |
| --- | --- | --- | --- | --- | --- | --- | --- | --- | --- | --- |
|  |  | Epilepsy cohort | Psychosis cohort | Depression cohort | Facility study | Community study |  | Health organisation | Community | Health facility |
| 2013 | 1 |  |  |  |  |  |  |  |  |  |
|  |  |  |  |  |  |  |  |  |  |  |
|  |  |  |  |  |  |  |  |  |  |  |
|  | 2 |  |  |  |  |  |  |  |  |  |
|  |  |  |  |  |  |  |  |  |  |  |
|  |  |  |  |  |  |  |  |  |  |  |
|  | 3 |  |  |  |  |  |  |  |  |  |
|  |  |  |  |  |  |  |  |  |  |  |
|  |  |  |  |  |  |  |  |  |  |  |
|  | 4 |  |  |  |  |  |  |  |  |  |
|  |  |  |  |  |  |  |  |  |  |  |
|  |  |  |  |  |  |  |  |  |  |  |
| 2014 | 1 |  |  |  |  |  |  |  |  |  |
|  |  |  |  |  |  |  |  |  |  |  |
|  |  |  |  |  |  |  |  |  |  |  |
|  | 2 |  |  |  |  |  |  |  |  |  |
|  |  |  |  |  |  |  |  |  |  |  |
|  |  |  |  |  |  |  |  |  |  |  |
|  | 3 |  |  |  |  |  |  |  |  |  |
|  |  |  |  |  |  |  |  |  |  |  |
|  |  |  |  |  |  |  |  |  |  |  |
|  | 4 |  |  |  |  |  |  |  |  |  |
|  |  |  |  |  |  |  |  |  |  |  |
|  |  |  |  |  |  |  |  |  |  |  |
| 2015 | 1 |  |  |  |  |  |  |  |  |  |
|  |  |  |  |  |  |  |  |  |  |  |
|  |  |  |  |  |  |  |  |  |  |  |
|  | 2 |  |  |  |  |  |  |  |  |  |
|  |  |  |  |  |  |  |  |  |  |  |
|  |  |  |  |  |  |  |  |  |  |  |
|  | 3 |  |  |  |  |  |  |  |  |  |
|  |  |  |  |  |  |  |  |  |  |  |
|  |  |  |  |  |  |  |  |  |  |  |
|  | 4 |  |  |  |  |  |  |  |  |  |
|  |  |  |  |  |  |  |  |  |  |  |
|  |  |  |  |  |  |  |  |  |  |  |
| 2016 | 1 |  |  |  |  |  |  |  |  |  |
|  |  |  |  |  |  |  |  |  |  |  |
|  |  |  |  |  |  |  |  |  |  |  |
|  | 2 |  |  |  |  |  |  |  |  |  |
|  |  |  |  |  |  |  |  |  |  |  |
|  |  |  |  |  |  |  |  |  |  |  |
|  | 3 |  |  |  |  |  |  |  |  |  |
|  |  |  |  |  |  |  |  |  |  |  |
|  |  |  |  |  |  |  |  |  |  |  |
|  | 4 |  |  |  |  |  |  |  |  |  |
|  |  |  |  |  |  |  |  |  |  |  |
|  |  |  |  |  |  |  |  |  |  |  |
| 2017 | 1 |  |  |  |  |  |  |  |  |  |
|  |  |  |  |  |  |  |  |  |  |  |
|  |  |  |  |  |  |  |  |  |  |  |
|  | 2 |  |  |  |  |  |  |  |  |  |
|  |  |  |  |  |  |  |  |  |  |  |
|  |  |  |  |  |  |  |  |  |  |  |
|  | 3 |  |  |  |  |  |  |  |  |  |
|  |  |  |  |  |  |  |  |  |  |  |
|  |  |  |  |  |  |  |  |  |  |  |
|  | 4 |  |  |  |  |  |  |  |  |  |
|  |  |  |  |  |  |  |  |  |  |  |
|  |  |  |  |  |  |  |  |  |  |  |

**Figure S2: Consort diagram – treatment cohort studies for mental health patients of primary health care centers in Kamuli District, Uganda.**

Recruitment from 13 implementation facilities in Kamuli district

Recruited and baseline completed (n=181)

Lost to follow-up (n=17)

- Relocation (n=6)
- Sickness/death (n=8)
- Not at home (n=2)
- Refusal (n=1)

All participants included in the analysis

- Baseline (n=181)
- Midline (n=174)
- Endline (n=162)

Diagnosed with epilepsy (n=181)

Midline assessment completed (n=174)

Endline assessment completed (n=162)

Recruited and baseline completed (n=51)

Lost to follow-up (n=8)

- Relocation (n=5)
- Not at home (n=1)
- Other reason (n=2)

All participants included in the analysis

- Baseline (n=51)
- Midline (n=48)
- Endline (n=43)

Diagnosed with psychosis (n=51)

Midline assessment completed (n=48)

Endline assessment completed (n=43)

Diagnosed with depression (n=64)

Recruited and baseline completed (n=64)

Lost to follow-up (n=7)

- Relocation (n=6)
- Death (n=1)

Midline assessment completed (n=61)

Endline assessment completed (n=57)

All participants included in the analysis

- Baseline (n=64)
- Midline (n=61)
- Endline (n=57)

**Table S1. Inequality of contact with a primary health care provider for adults with probable depression in Kamuli District, Uganda, 2017.**

| Characteristic | | Contact with primary care provider n, (%) | Chi-square P |
| --- | --- | --- | --- |
| Overall | | 94/452 (19.4) |  |
|  | |  |  |
| By sex | |  |  |
|  | Men | 14/101 (13.6) | 0.046 |
|  | Women | 87/351 (23.3) |  |
|  |  |  |  |
| By education | |  |  |
|  | Less than primary | 24/95 (23.8) | 0.781 |
|  | Primary | 60/262 (20.4) |  |
|  | Secondary or more | 17/95 (19.9) |  |

Counts are reported as observed, while proportions and P-values are design-adjusted for the population-based survey design

**Table S2. Inequality of clinical detection of depression and of alcohol use disorder among adult outpatients in PRIME implementation clinics in Kamuli, Uganda, 2014 & 2017.**

| Characteristic | |  | Midline | |  | Endline | |
| --- | --- | --- | --- | --- | --- | --- | --- |
|  | Stratum |  | Clinical detection (%) | Fisher’s Exact P |  | Clinical detection (%) | Fisher’s Exact P |
| Depression | |  |  | |  |  | |
| Overall | |  | 19/149 (12.7) |  |  | 5/103 (4.8) |  |
| Sex | |  |  |  |  |  |  |
|  | Men |  | 3/34 (8.8) | 0.57 |  | 3/31 (9.7) | 0.16 |
|  | Women |  | 16/115 (13.9) |  |  | 2/72 (2.8) |  |
|  |  |  |  |  |  |  |  |
| By education | |  |  |  |  |  |  |
|  | Less than primary |  | 12/54 (22.2) | 0.03 |  | 1/44 (2.3) | 0.05 |
|  | Primary |  | 5/77 (6.5) |  |  | 1/42 (2.4) |  |
|  | Secondary or more |  | 2/18 (11.1) |  |  | 3/17 (17.6) |  |
|  | |  |  |  |  |  |  |
| Alcohol use disorder | |  |  | |  |  | |
| Overall | |  | 4/32 (12.5) |  |  | 2/38 (5.3) |  |
| By sex | |  |  |  |  |  |  |
|  | Men |  | 3/29 (10.3) | 0.34 |  | 1/27 (3.7) | 0.50 |
|  | Women |  | 1/3 (33.3) |  |  | 1/11 (9.1) |  |
|  |  |  |  |  |  |  |  |
| By education | |  |  |  |  |  |  |
|  | Less than primary |  | 1/10 (10.0) | 0.99 |  | 1/17 (5.9) | 0.99 |
|  | Primary |  | 2/12 (16.7) |  |  | 1/13 (7.7) |  |
|  | Secondary or more |  | 1/10 (10.0) |  |  | 0/8 (0.0) |  |

**Table S3. Inequity of change in symptom severity and impairment score for depression, epilepsy and psychosis patients at PRIME implementation clinics in Kamuli, Uganda, 2015-2017.**

| Outcome | | |  | Midline ^a^ versus baseline | |  | Endline versus baseline | |
| --- | --- | --- | --- | --- | --- | --- | --- | --- |
|  | Stratum | |  | Difference (95% CI) | P-value ^b^ |  | Difference (95% CI) | P-value ^b^ |
| Depression | | |  |  |  |  |  |  |
| Symptom severity (Mean PHQ-9) | | |  |  |  |  |  |  |
|  | Overall | |  | -7.9 (-12.6 to -3.2) |  |  | -9.9 (-14.4 to -5.4) |  |
|  | Sex | |  |  |  |  |  |  |
|  |  | Men |  | -8.2 (-17.9 to 1.6) | 0.95 |  | -8.8 (-18.5 to 0.9) | 0.80 |
|  |  | Women |  | -7.8 (-13.2 to -2.5) |  |  | -10.2 (-15.3 to -5.2) |  |
|  | Education | |  |  |  |  |  |  |
|  |  | Less than primary school |  | -6.2 (-16.5 to 4.1) | 0.93 |  | -8.9 (-18.5 to 0.6) | 0.97 |
|  |  | Secondary school achieved |  | -8.5 (-14.4 to -2.6) |  |  | -10.3 (-16.1 to -4.6) |  |
|  |  | Tertiary education achieved |  | -8.3 (-19.5 to 2.9) |  |  | -10.0 (-20.8 to 0.7) |  |
|  |  |  |  |  |  |  |  |  |
| Impairment (Mean WHODAS) | | |  |  |  |  |  |  |
|  | Overall | |  | -21.6 (-35.4 to -7.9) |  |  | -25.8 (-39.2 to -12.4) |  |
|  | Sex | |  |  |  |  |  |  |
|  |  | Men |  | -27.1 (-56.8 to 2.6) | 0.67 |  | -29.4 (-35.4 to -4.4) | 0.77 |
|  |  | Women |  | -19.9 (-35.4 to -4.4) |  |  | -24.5 (-39.5 to -9.6) |  |
|  | Education | |  |  |  |  |  |  |
|  |  | Less than primary school |  | -7.2 (-39.7 to 25.2) | 0.44 |  | -18.7 (-47.7 to 10.3) | 0.72 |
|  |  | Secondary school achieved |  | -23.0 (-40.4 to -5.6) |  |  | -25.5 (-42.7 to -8.3) |  |
|  |  | Tertiary education achieved |  | -36.4 (-67.0 to -5.7) |  |  | -36.1 (-66.8 to -5.4) |  |
| Psychosis | | |  |  |  |  |  |  |
| Impairment (Mean WHODAS) | | |  |  |  |  |  |  |
|  | Overall | |  | -20.7 (-31.3 to -10.1) |  |  | -19.2 (-30.1 to -8.4) |  |
|  | Sex | |  |  |  |  |  |  |
|  |  | Men |  | -13.4 (-27.6 to 0.8) | 0.14 |  | -13.0 (-27.5 to 1.4) | 0.22 |
|  |  | Women |  | -29.6 (-45.8 to -13.3) |  |  | -26.9 (-43.6 to -10.2) |  |
|  | Education | |  |  |  |  |  |  |
|  |  | Illiterate |  | -2.8 (-23.7 to 8.2) | 0.32 |  | 13.9 (-33.8 to 61.6) | 0.34 |
|  |  | Less than primary school achieved |  | -20.6 (-36.2 to -5.1) |  |  | -16.4 (-32.7 to -0.0) |  |
|  |  | Primary school or higher achieved |  | -21.1 (-36.2 to -6.0) |  |  | -22.7 (-37.7 to -7.7) |  |
|  |  |  |  |  |  |  |  |  |
| Epilepsy | | |  |  |  |  |  |  |
| Symptom severity (Median # seizures/30 days) | | | | |  |  |  |  |
|  | Overall | |  | -1 (-3 to 1) |  |  | -1 (-3 to 1) |  |
|  | Sex | |  |  |  |  |  |  |
|  |  | Men |  | -1 (-3 to 1) | 0.89 ^c^ |  | -1 (-3 to 0) | 0.42 ^c^ |
|  |  | Women |  | -1 (-4 to 1) |  |  | -1 (-3 to 2) |  |
|  | Education | |  |  |  |  |  |  |
|  |  | Less than primary school |  | -1 (-4 to 2) | 0.26 ^d^ |  | -1 (-4 to 2) | 0.97 ^d^ |
|  |  | Secondary school achieved |  | -1 (-2.5 to 0.5) |  |  | -1 (-3 to 1) |  |
|  |  | Tertiary education achieved |  | 0 (-1 to 13) |  |  | -0.5 (-2 to 1) |  |
|  |  |  |  |  |  |  |  |  |
| Impairment (Mean WHODAS) | | |  |  |  |  |  |  |
|  | Overall | |  | -9.1 (-17.4 to -0.8) |  |  | -12.4 (-20.5 to -4.4) |  |
|  | Sex | |  |  |  |  |  |  |
|  |  | Men |  | -8.5 (-20.4 to 3.3) | 0.90 |  | -18.4 (-29.0 to -7.8) | 0.14 |
|  |  | Women |  | -9.7 (-21.2 to 1.9) |  |  | -6.4 (-18.5 to 5.8) |  |
|  | Education | |  |  |  |  |  |  |
|  |  | Less than primary school |  | -8.9 (-24.8 to 7.1) | 0.83 |  | -13.6 (-28.7 to 1.5) | 0.98 |
|  |  | Secondary school achieved |  | -10.4 (-20.8 to 0.0) |  |  | -12.2 (-22.5 to -1.8) |  |
|  |  | Tertiary education achieved |  | -1.7 (-27.8 to 24.3) |  |  | -10.7 (-33.8 to 12.4) |  |

^a^ 3 months for depression, 6 months for psychosis and epilepsy

^b^ P-value from the Wald test of interaction terms in a negative binomial regression model unless otherwise indicated
